# Supplementary material for: Characterization of Cytokine Treatment on Human Pancreatic Islets by Top‐Down Proteomics
Source: Proteomics. 2025 Sep 21;25(24):55–66. doi: 10.1002/pmic.70044 (PMC12716117; doi:10.1002/pmic.70044)
Supplement: Supplementary file 2 — Supporting File 2: pmic70044‐sup‐0002‐SuppMat.docx [file PMIC-25--s002.docx]

**Supplementary Information for**

**Characterization of cytokine treatment on human pancreatic islets by top-down proteomics**

Ashley N Ives^1^, Tyler Sagendorf^2^, Lorenz Nierves^2^, Tai-Tu Lin^2^, Ercument Dirice^3^, Rohit N Kulkarni^4-6^, Ljiljana Paša-Tolić^1^, Wei-Jun Qian^2*^, James M Fulcher^1*^

^1^Environmental Molecular Sciences Laboratory, Pacific Northwest National Laboratory, Richland, WA 99354, USA

^2^Biological Sciences Division, Pacific Northwest National Laboratory, Richland, WA 99354, USA

^3^Department of Pharmacology and Medicine, New York Medical College School of Medicine, Valhalla, NY 10595, USA

^4^Islet Cell and Regenerative Biology, Joslin Diabetes Center, Boston, MA 02215, USA

^5^Department of Medicine, Beth Israel Deaconess Medical Center, Harvard Medical School, Boston, MA 02215, USA

^6^Harvard Stem Cell Institute, Harvard Medical School, Boston, MA 02215, USA

*Correspondence: weijun.qian@pnnl.gov (W.Q.) & [james.fulcher@pnnl.gov](mailto:james.fulcher@pnnl.gov) (J.M.F.)

Contents

[**Table S1. Patient meta data for islet donors.** 3](#_Toc206491022)

[**Figure S1. Histogram of monoisotopic masses for observed proteoforms.** 4](#_Toc206491023)

[**Figure S2. Histogram of relative standard deviations (RSD) based on label-free, intensity-based quantification of observed proteoforms.** 5](#_Toc206491024)

[**Figure S3. Fragmentation maps of select insulin (INS) proteoforms.** 6](#_Toc206491025)

[**Figure S4. Summary of all observed IAPP proteoforms.** 7](#_Toc206491026)

[**Figure S5. Summary of top 30 most abundant SST proteoforms.** 8](#_Toc206491027)

[**Figure S6. Summary of top 30 most abundant PPY proteoforms.** 9](#_Toc206491028)

[**Figure S7. Fragmentation maps of glycosylated PPY.** 10](#_Toc206491029)

[**Figure S8. Summary of top 30 most abundant CHGA proteoforms.** 11](#_Toc206491030)

[**Figure S9. Summary of top 30 most abundant CHGB/SCG1 proteoforms.** 12](#_Toc206491031)

[**Figure S10. Summary of all observed CHGC/SCG2 proteoforms.** 13](#_Toc206491032)

[**Figure S11. Summary of top 30 most abundant VGF proteoforms.** 14](#_Toc206491033)

[**Figure S12. Fragmentation map for C-terminally amidated, oxidized GCG_92-127_.** 15](#_Toc206491034)

[**Figure S13. Proteoforms of HMGN1 and HMGN2.** 16](#_Toc206491035)

# **Table S1. Patient meta data for islet donors.**

Patient meta data for islet donors. Each patient is assigned a United Network for Organ Sharing (UNOS) donor identity. Data is given for the donor age (years), sex, ethnicity, body mass index (BMI), and glycated hemoglobin (HgbA1c,%).

| **UNOS Donor Identity** | **Age (years)** | **Sex** | **Ethnicity** | **BMI** | **HgbA1c (%)** |
| --- | --- | --- | --- | --- | --- |
| ADC5225A | 27 | M | Hispanic | 30.7 | 5.2 |
| ADC4231 | 29 | M | White | 32.5 | 5.5 |
| ADIL276 | 51 | F | Hispanic | 30.1 | NA |
| ADIR339 | 44 | F | White | 34.5 | NA |
| ADH3495 | 53 | F | Hispanic | 33.3 | 6 |
| ADH1303 | 50 | F | White | 20.3 | NA |


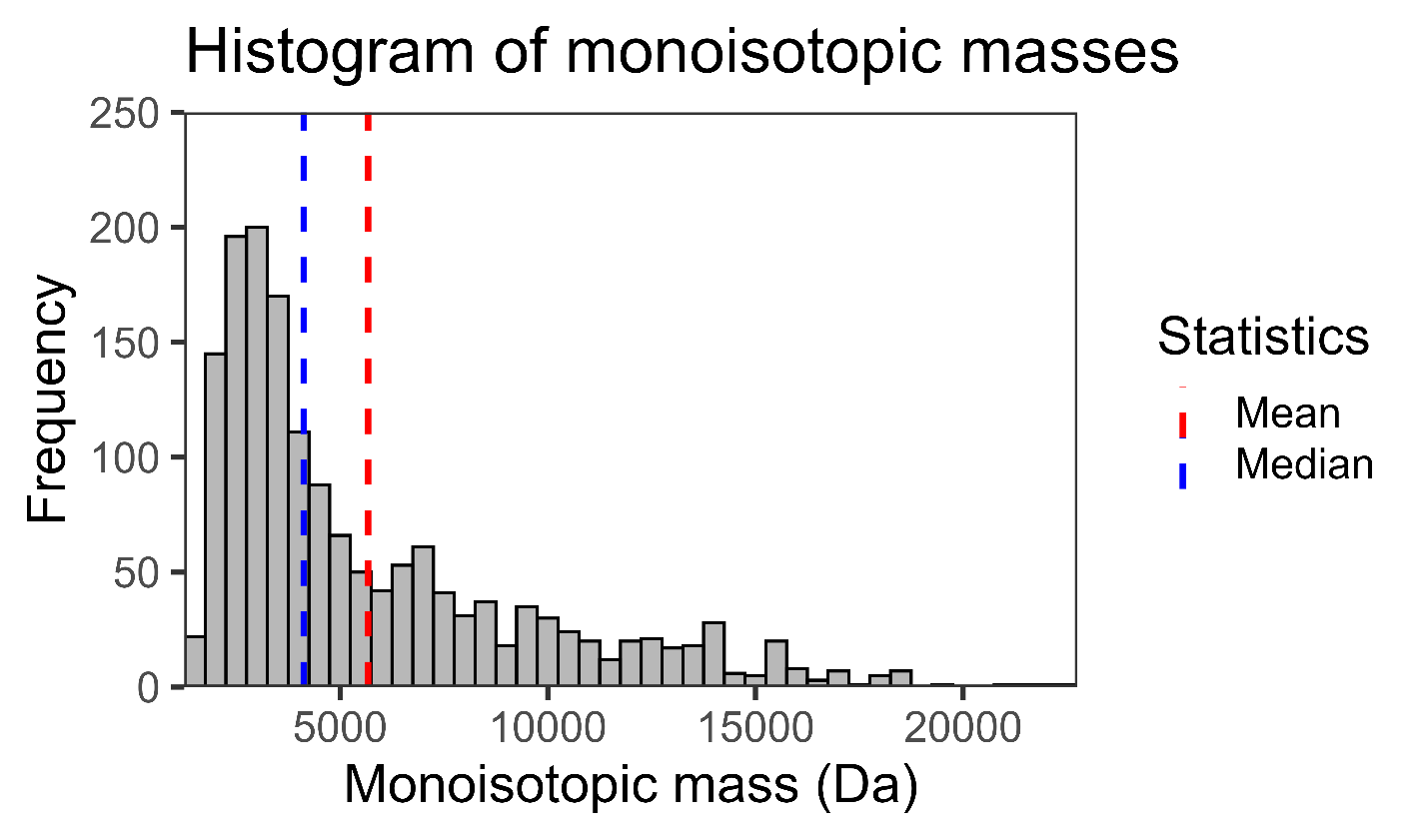


# **Figure S1. Histogram of monoisotopic masses for observed proteoforms.**

Histogram of monoisotopic masses (Da) for observed proteoforms; data is shown for all donors and treatment conditions (N=6, t=2). The mean (red) and median (blue) monoisotopic proteoform mass (Da) are annotated as dashed lines.


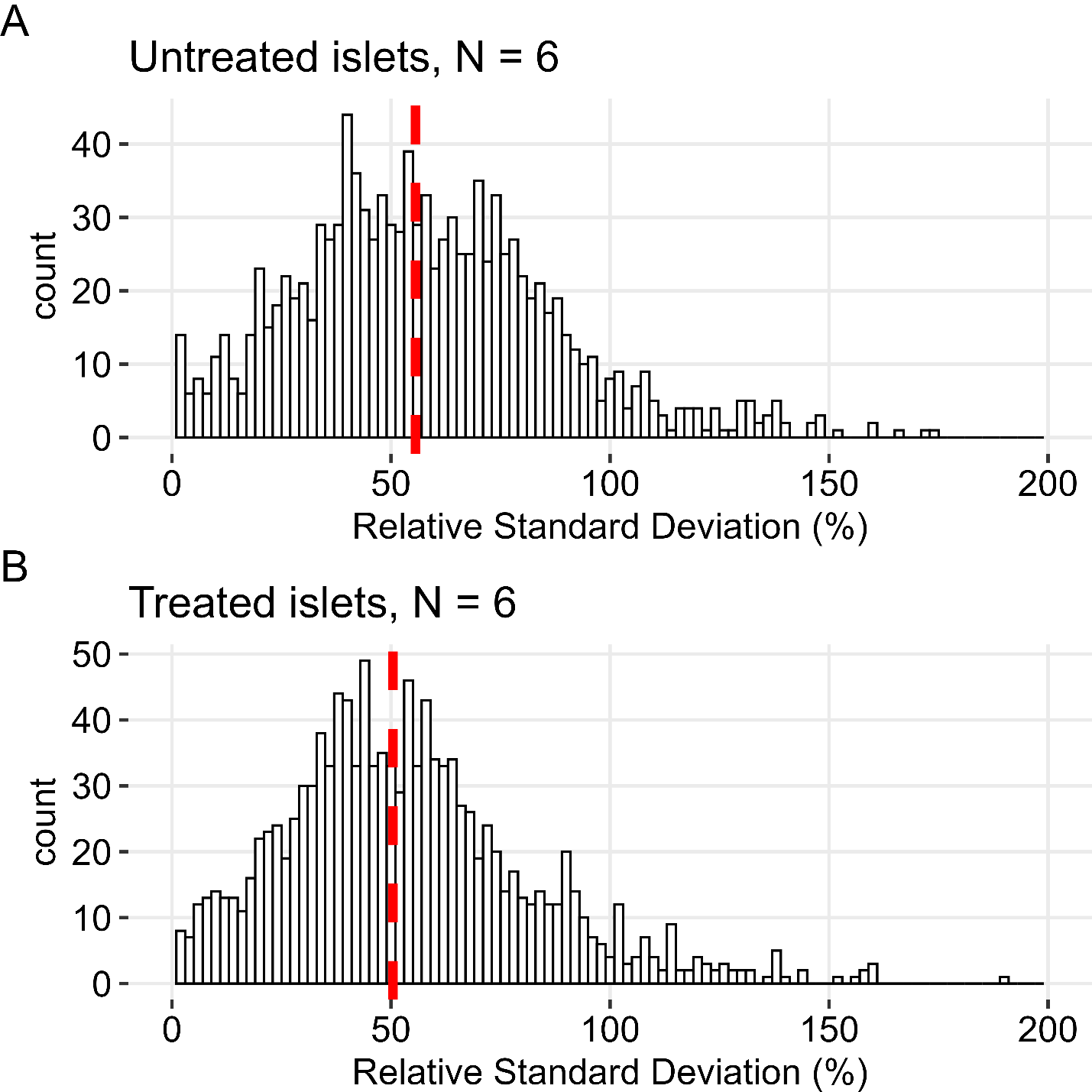


# **Figure S2. Histogram of relative standard deviations (RSD) based on label-free, intensity-based quantification of observed proteoforms.**

Histogram of relative standard deviations (RSDs) for observed proteoforms. RSDs were calculated using log2(label-free intensities) following median normalization. RSDs are shown for (A) untreated, control islets and (B) islets after cytokine treatment. The median (red) RSD for a given condition is annotated as a dashed line.


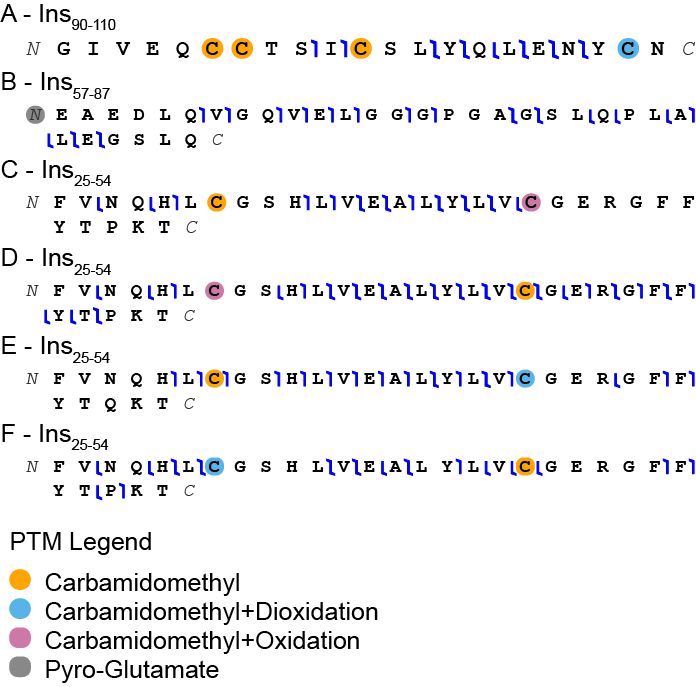


# **Figure S3. Fragmentation maps of select insulin (INS) proteoforms.**

Maps denote matching fragment ions for searched candidate proteoforms including (A) dioxidation of Cys108, (B) pyro-glutamylation of the C peptide, oxidation within the B chain localized to either (C) Cys43 or (D) Cys31, and dioxidation within the B chain localized to either (E) Cys43 or (F) Cys31. Note that panels (C) and (D) are derived from the same proteoform spectral match scan. (E) and (F) are also derived from the same proteoform spectral match scan. Colored circles denote searched modifications including carbamidomethyl (gold), carbamidomethyl and dioxidation (blue), carbamidomethyl and oxidation (pink), and pyroglutamate (gray). Panel labels denote the first and last amino acid of a searched proteoform (i.e. Ins_firstAA-lastAA_). Blue flags denote either *b*- (left-facing) or *y*-type (right-facing) fragment ions. The N- and C-termini are denoted as italized letters.


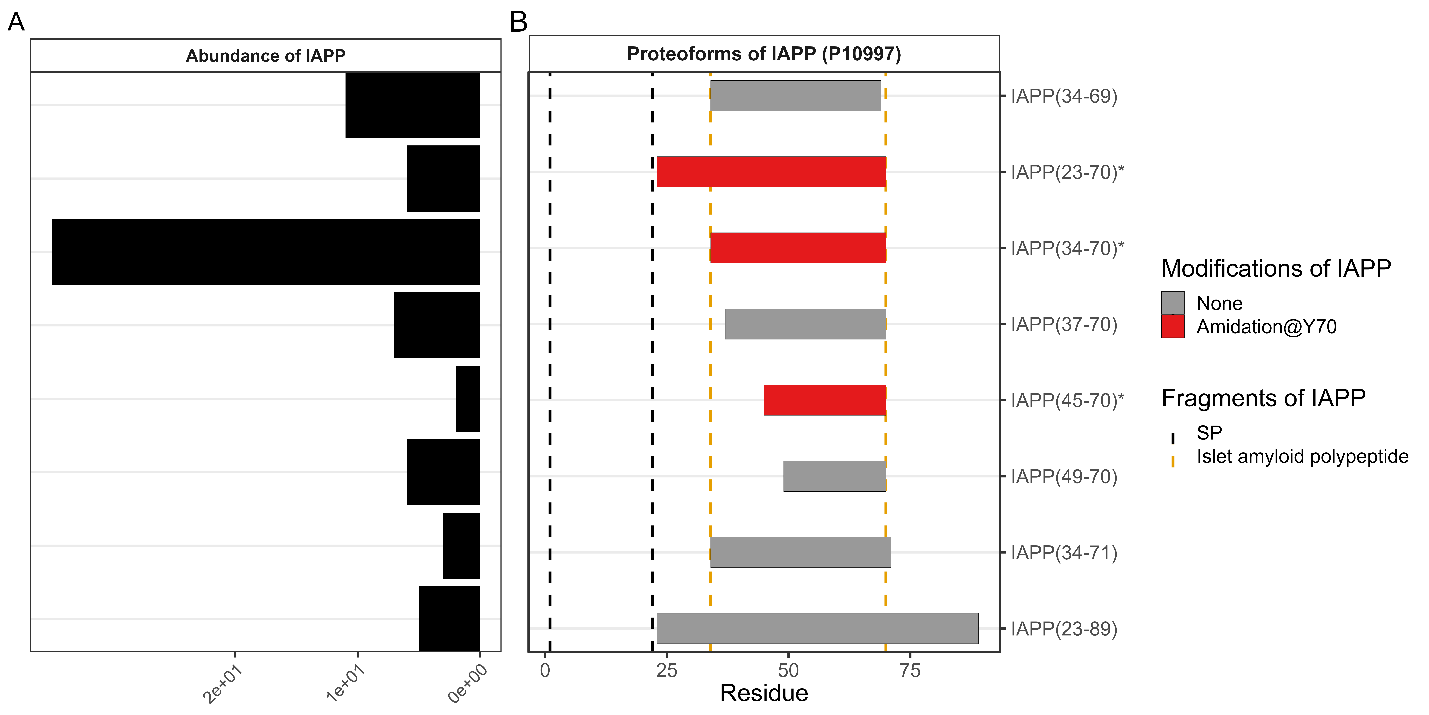


# **Figure S4. Summary of all observed IAPP proteoforms.**

(A) Median spectral count abundance for (B) islet amyloid polypeptide (IAPP) proteoforms. The rightmost panel maps the first and last amino acid of a given proteoform (x-axis), and color fill denotes identified PTMs. Dashed vertical lines annotate the region of a given gene. Proteoforms are sorted top to bottom by ascending C-terminal amino acid ending position, followed by ascending N-terminal amino acid starting position. Y-axis labels denote the first and last amino acid of a given proteoform, and “*” is used to denote modified proteoforms.


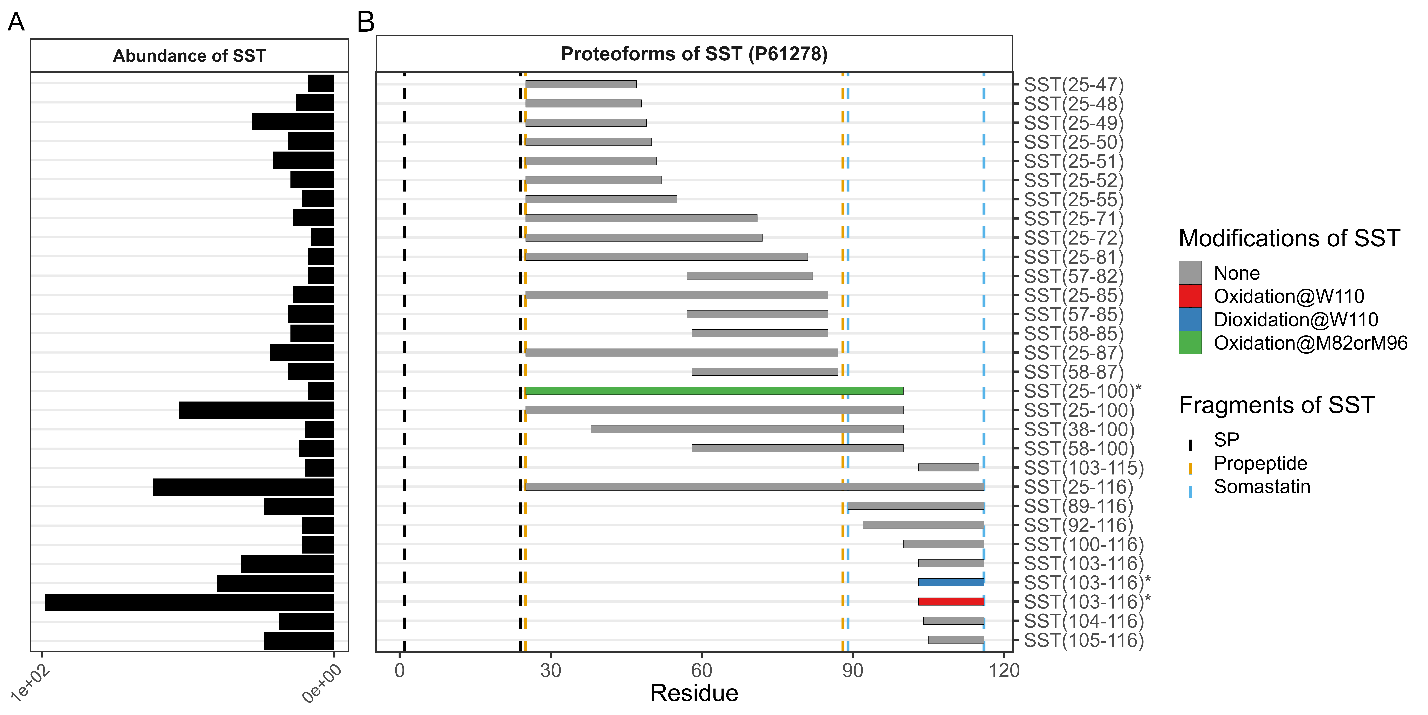


# **Figure S5. Summary of top 30 most abundant SST proteoforms.**

(A) Median spectral count abundance for (B) somatostatin (SST) proteoforms. The rightmost panel maps the first and last amino acid of a given proteoform (x-axis), and color fill denotes identified PTMs. Dashed vertical lines annotate the region of a given gene. Proteoforms are sorted top to bottom by ascending C-terminal amino acid ending position, followed by ascending N-terminal amino acid starting position. Y-axis labels denote the first and last amino acid of a given proteoform, and “*” is used to denote modified proteoforms.


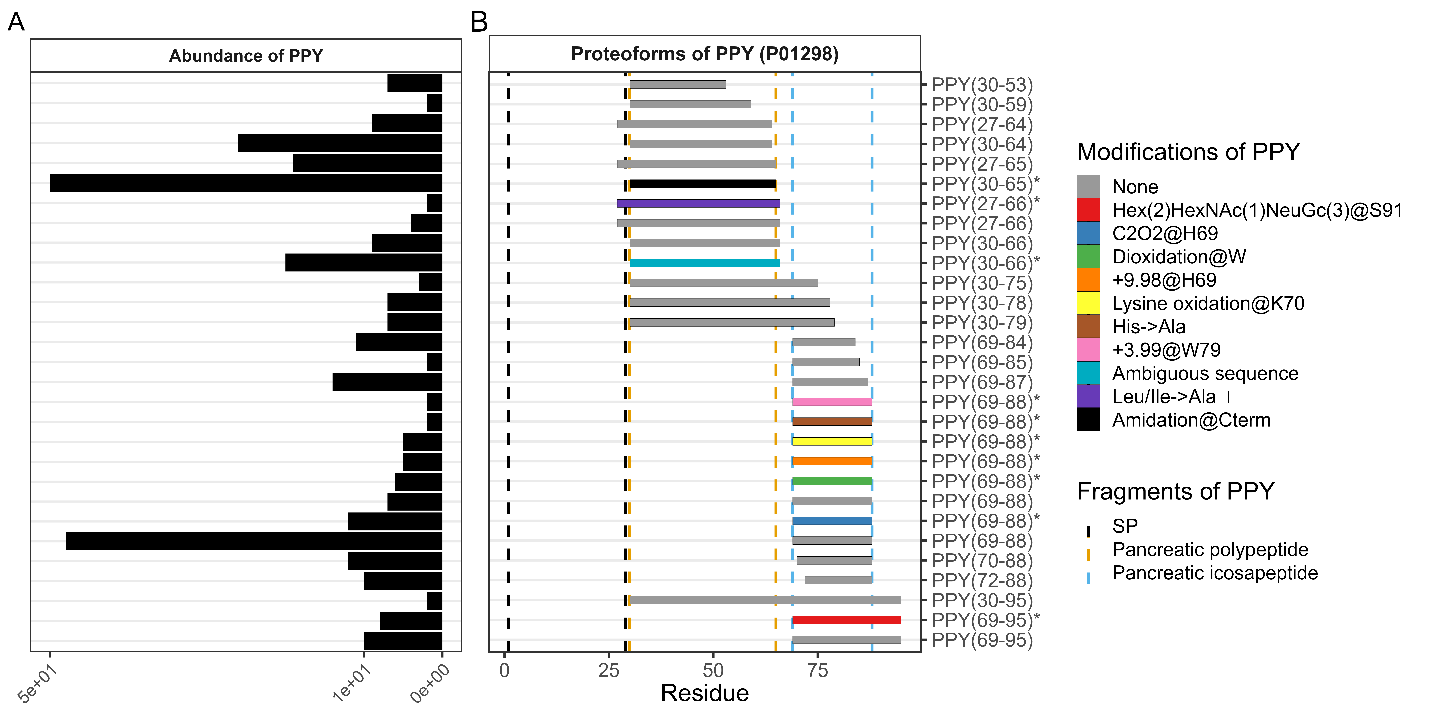


# **Figure S6. Summary of top 30 most abundant PPY proteoforms.**

(A) Median spectral count abundance for (B) pancreatic polypeptide prohormone (PPY) proteoforms. The rightmost panel maps the first and last amino acid of a given proteoform (x-axis), and color fill denotes identified PTMs. Dashed vertical lines annotate the region of a given gene. Proteoforms are sorted top to bottom by ascending C-terminal amino acid ending position, followed by ascending N-terminal amino acid starting position. Y-axis labels denote the first and last amino acid of a given proteoform, and “*” is used to denote modified proteoforms. “Ambiguous sequence” denotes a level 4 proteoform that could be assigned to either canonical PPY (SwissProt: P01298) or a PPY TrEMBL annotation (TrEMBL: K7EKP2).


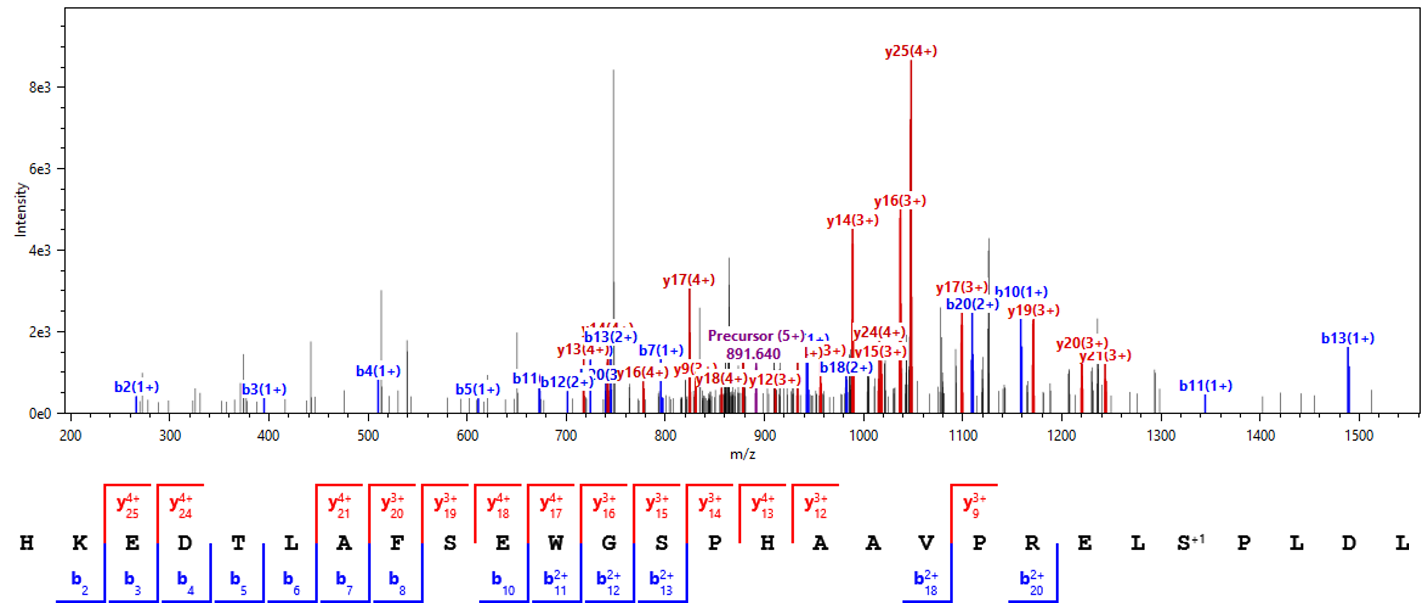


# **Figure S7. Fragmentation maps of glycosylated PPY.**

(Top) Annotated fragmentation spectrum of b- (blue) and y- (red) type fragment ions matching the candidate (Bottom) PPY sequence. The matched precursor peak is highlighted in purple. Matched fragment ions are mapped on the PPY proteoform sequence as blue or red flags for b- or y-type fragment ions, respectively. “S+1” denotes where the +1448.66 is placed in the search.


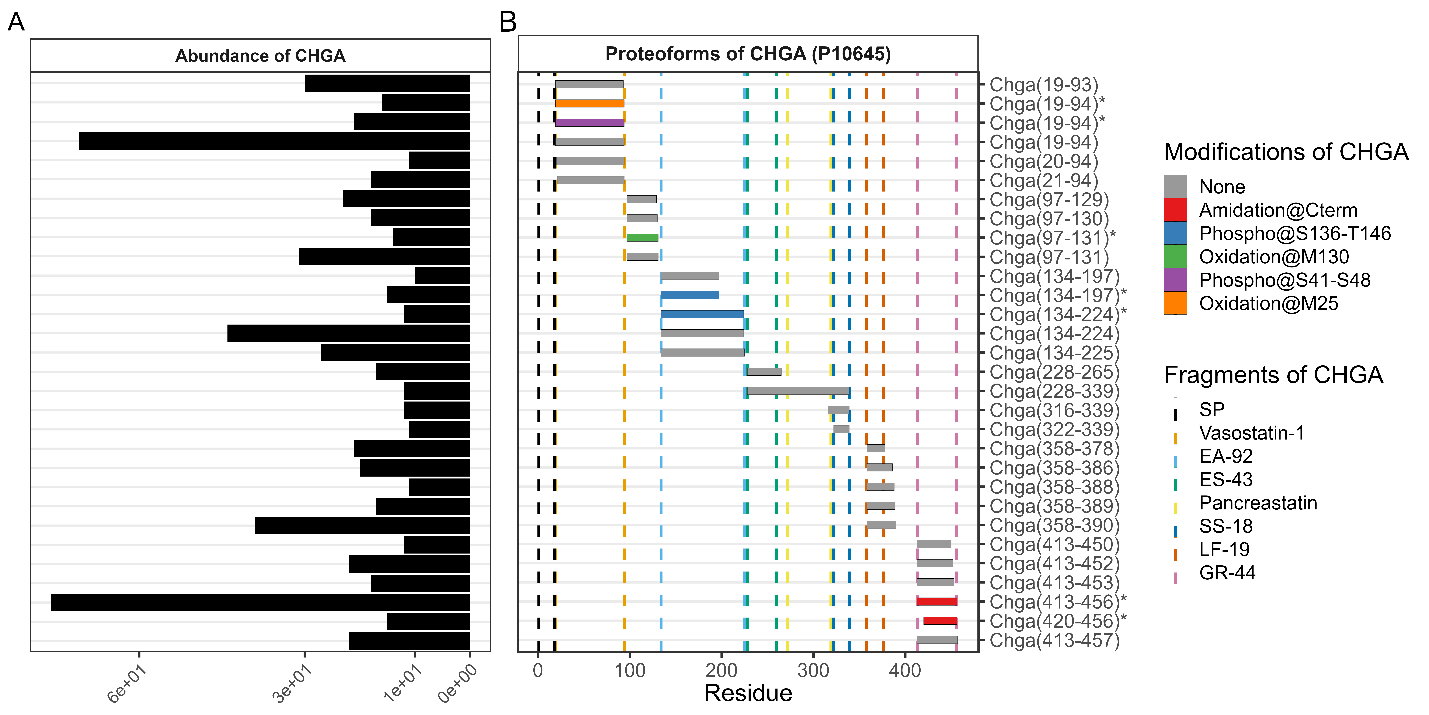


# **Figure S8. Summary of top 30 most abundant CHGA proteoforms.**

(A) Median spectral count abundance for (B) chromogranin-A (CHGA) proteoforms. The rightmost panel maps the first and last amino acid of a given proteoform (x-axis), and color fill denotes identified PTMs. Dashed vertical lines annotate the region of a given gene. Proteoforms are sorted top to bottom by ascending C-terminal amino acid ending position, followed by ascending N-terminal amino acid starting position. Y-axis labels denote the first and last amino acid of a given proteoform, and “*” is used to denote modified proteoforms.


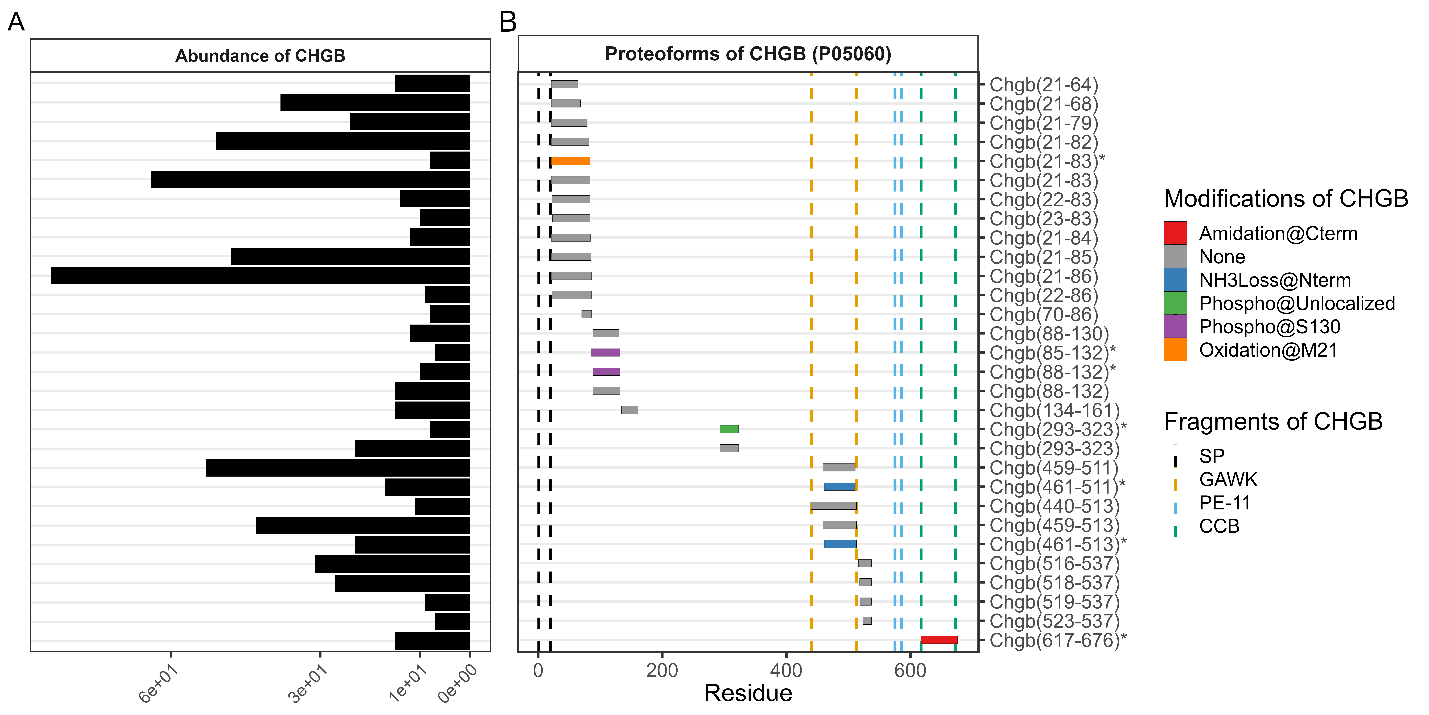


# **Figure S9. Summary of top 30 most abundant CHGB/SCG1 proteoforms.**

(A) Median spectral count abundance for (B) chromogranin-B (CHGB)/ secretogranin-1 (SCG1) proteoforms. The rightmost panel maps the first and last amino acid of a given proteoform (x-axis), and color fill denotes identified PTMs. Dashed vertical lines annotate the region of a given gene. Proteoforms are sorted top to bottom by ascending C-terminal amino acid ending position, followed by ascending N-terminal amino acid starting position. Y-axis labels denote the first and last amino acid of a given proteoform, and “*” is used to denote modified proteoforms.


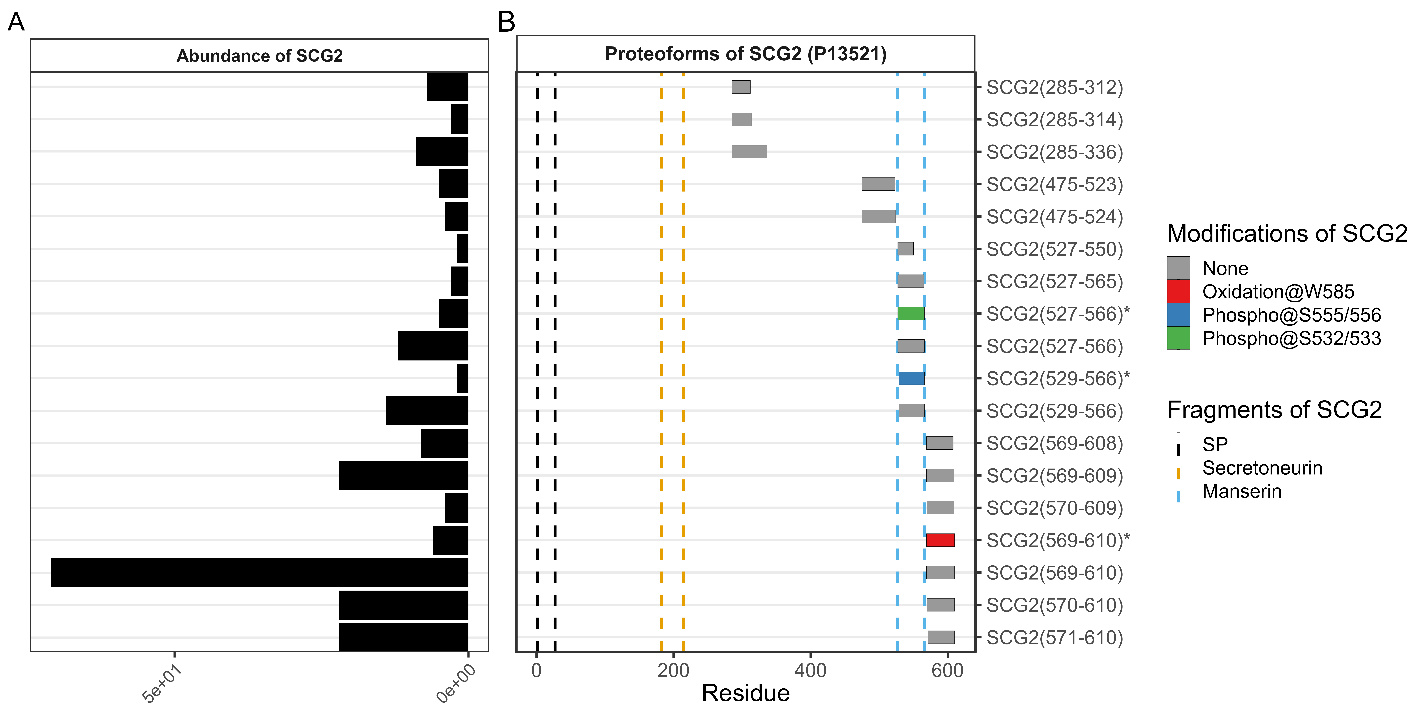


# **Figure S10. Summary of all observed CHGC/SCG2 proteoforms.**

(A) Median spectral count abundance for (B) chromogranin-C (CHGC)/ secretogranin-2 (SCG2) proteoforms. The rightmost panel maps the first and last amino acid of a given proteoform (x-axis), and color fill denotes identified PTMs. Dashed vertical lines annotate the region of a given gene. Proteoforms are sorted top to bottom by ascending C-terminal amino acid ending position, followed by ascending N-terminal amino acid starting position. Y-axis labels denote the first and last amino acid of a given proteoform, and “*” is used to denote modified proteoforms.


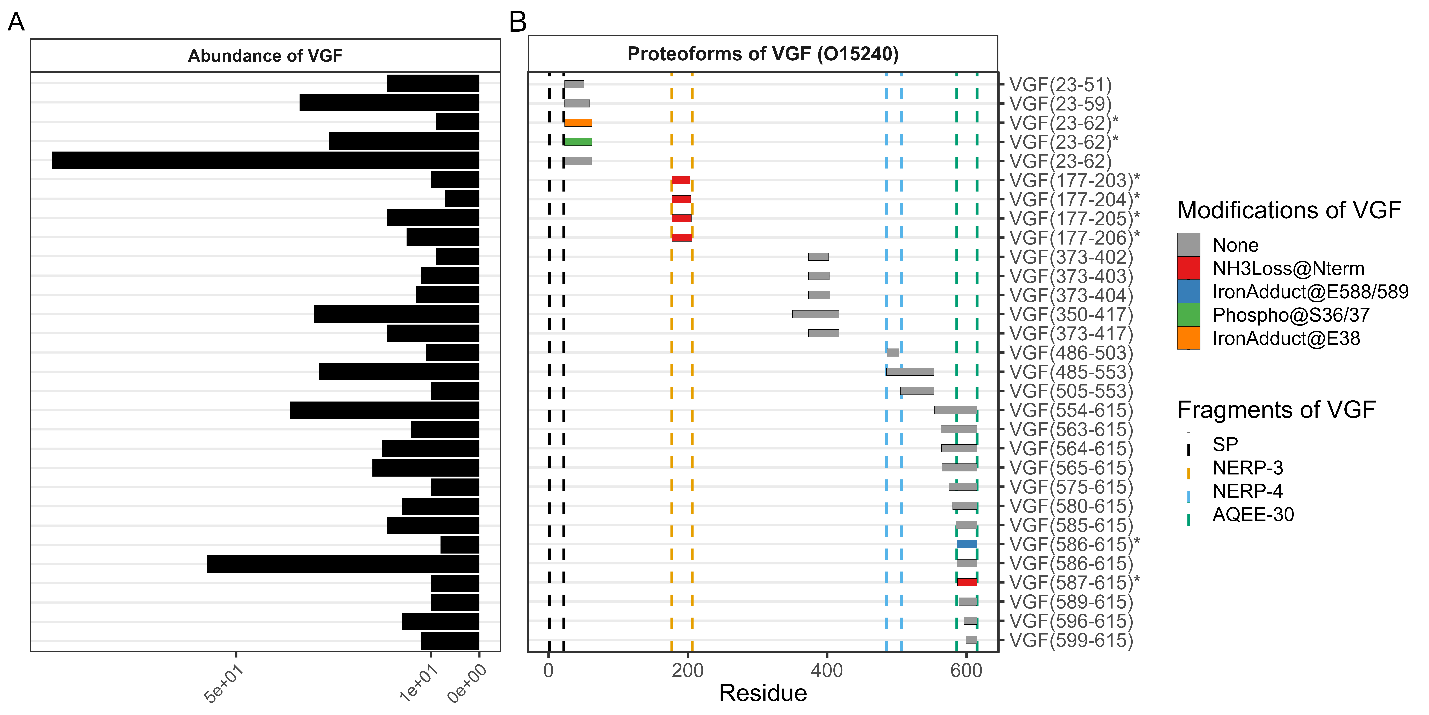


# **Figure S11. Summary of top 30 most abundant VGF proteoforms.**

(A) Median spectral count abundance for (B) VGF proteoforms. The rightmost panel maps the first and last amino acid of a given proteoform (x-axis), and color fill denotes identified PTMs. Dashed vertical lines annotate the region of a given gene. Proteoforms are sorted top to bottom by ascending C-terminal amino acid ending position, followed by ascending N-terminal amino acid starting position. Y-axis labels denote the first and last amino acid of a given proteoform, and “*” is used to denote modified proteoforms.


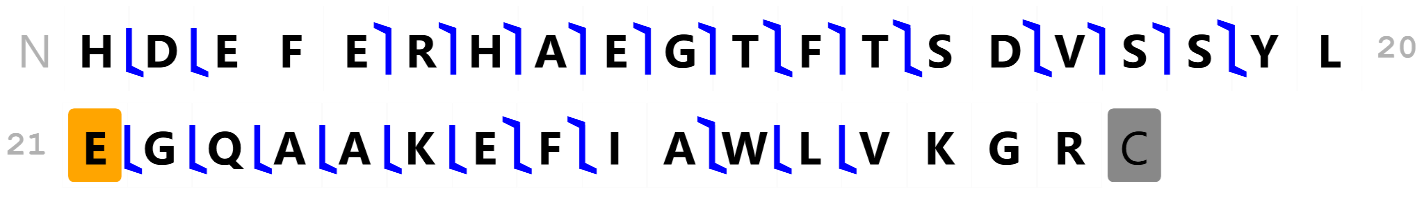


# **Figure S12. Fragmentation map for C-terminally amidated, oxidized GCG_92-127_.**

Assigned fragment ions are shown for the lowest (highest confidence) E-value (metric for how well experimental spectra matches a theoretical spectra or protein sequence databases) scan for C-terminally amidated, oxidized GCG_92-127_. The amidation site is denoted with a gray box. The gold box denotes a carbonyl modification (+O, -2H). Blue flags denote either *b*- (left-facing) or *y*-type (right-facing) fragment ions. The N- and C-termini are denoted as gray letters.


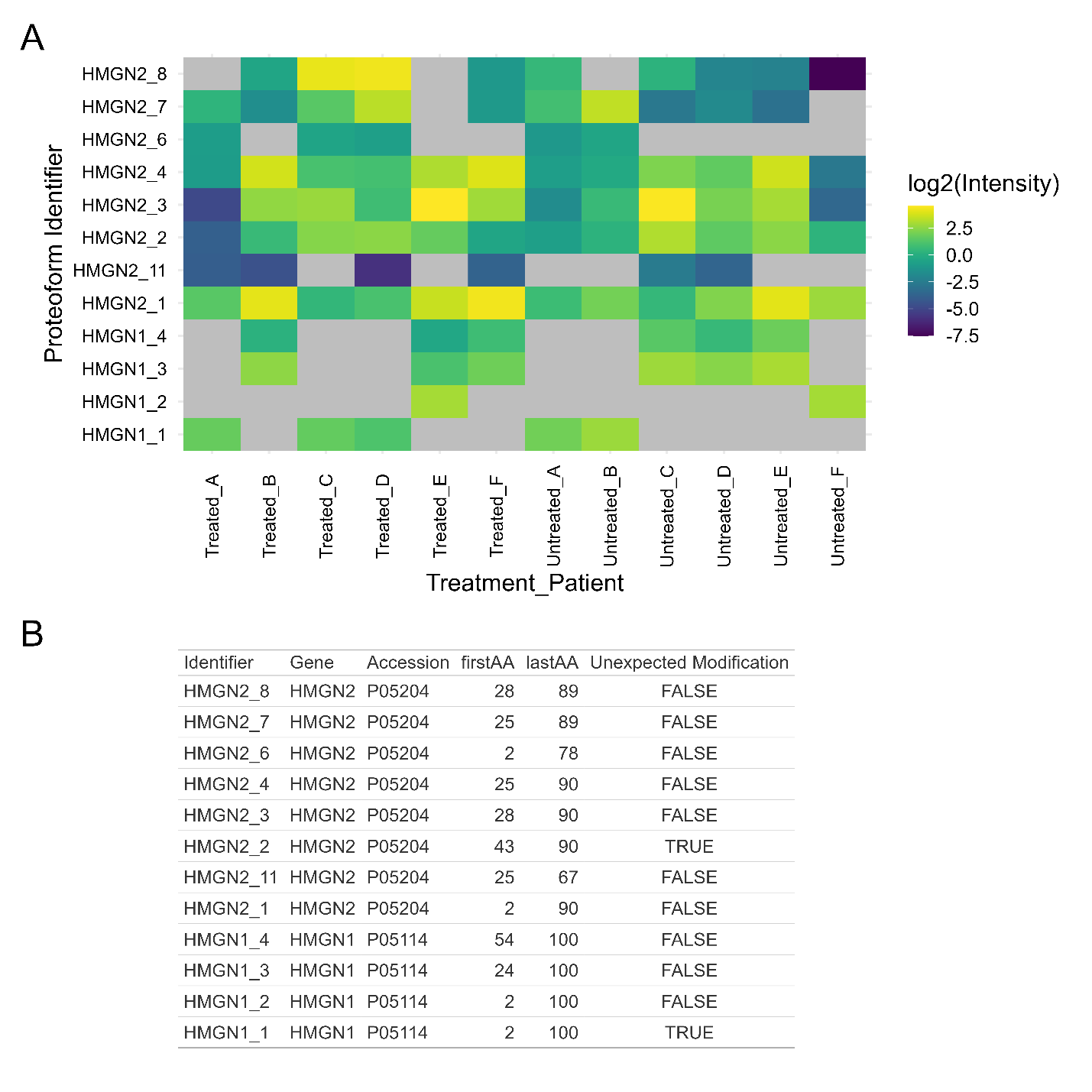


# **Figure S13. Proteoforms of HMGN1 and HMGN2.**

(A) Heatmap of HMGN1 and HMGN2 proteoforms. Proteoforms are listed as gene_ arbitrary proteoform identifiers on the y-axis. Patients are listed as treatment_patient identifier on the x-axis. Tile color denotes the median normalized log2(Intensity) as determined by label-free quantification. NA values are shown in gray. (B) Summarizes all proteoforms plotted in panel (A) including the first and last amino acid (firstAA, lastAA) based on the listed UniProt Accession. Proteoforms with unknown mass shifts are listed as “TRUE” in column “Unexpected Modification”.
